# Sphingolipids as Mediators in the Crosstalk between Microbiota and Intestinal Cells: Implications for Inflammatory Bowel Disease

**DOI:** 10.1155/2016/9890141

**Published:** 2016-08-30

**Authors:** Phillips-Farfán Bryan, Carvajal Karla, Medina-Torres Edgar Alejandro, Espinosa-Padilla Sara Elva, Fabrias Gemma, Camacho Luz

**Affiliations:** ^1^Laboratorio de Nutrición Experimental, Instituto Nacional de Pediatría, 04530 Ciudad de México, Mexico; ^2^Unidad de Investigación en Inmunodeficiencias, Instituto Nacional de Pediatría, 04530 Ciudad de México, Mexico; ^3^Research Unit on Bioactive Molecules, Department of Biomedicinal Chemistry, Institute for Advanced Chemistry of Catalonia (IQAC-CSIC), 08034 Barcelona, Spain

## Abstract

Inflammatory bowel disease (IBD) describes different illnesses characterized by chronic inflammation of the gastrointestinal tract. Although the pathogenic mechanisms leading to IBD are poorly understood, immune system disturbances likely underlie its development. Sphingolipids (SLs) have been identified as important players and promising therapeutic targets to control inflammation in IBD. Interestingly, it seems that microorganisms of the normal gut microbiota and probiotics are involved in sphingolipid function. However, there is a great need to investigate the role of SLs as intermediates in the crosstalk between intestinal immunity and microorganisms. This review focuses on recent investigations that describe some mechanisms involved in the regulation of cytokine profiles by SLs. We also describe the importance of gut microbiota in providing signaling molecules that favor the communication between resident bacteria and intestinal cells. This, in turn, modulates the immune response in the bowel and likely in other peripheral organs. The potential of SLs and gut microbiota as targets or therapeutic agents for IBD is also discussed.

## 1. Introduction

Inflammatory bowel disease (IBD) is a collection of digestive tract pathologies with chronic inflammation, such as Crohn's disease (CD) and ulcerative colitis (UC). Both conditions usually cause diarrhea, pain, fatigue, and weight loss, among other symptoms. CD causes inflammation in many parts of the gastrointestinal (GI) tract, whereas UC only affects the colon. Currently, the specific etiology of IBD is not well established. However, research in this field points to GI immune system dysfunction as a central pathogenic component [1]. The GI tract is continuously exposed to a great diversity of antigens from foods, bacteria, and parasites. Consequently, immunity in this organ is suppressed to avoid inflammation and maintain homeostasis. Any disruption in the regulatory mechanisms of the GI immune system leads to an excessive response that causes chronic inflammation [2, 3]. Recently, sphingolipids (SLs), particular membrane lipids, have been shown to play an important role in modulating the GI immune response and are promising therapeutic targets for IBD [4]. It has been also observed that the bacteria that naturally colonize the gut, named microbiota, may be involved in the metabolism of SLs, including their biosynthesis. Thus, microbiota and their interaction with probiotics likely are pivotal players in regulating GI immunity. The crosstalk between these components needs to be investigated more deeply. In this review, we will focus on the relationships between SLs, intestinal microbiota, and probiotics with a particular emphasis on their influence upon IBD.

## 2. Sphingolipids

**Figure 1:**
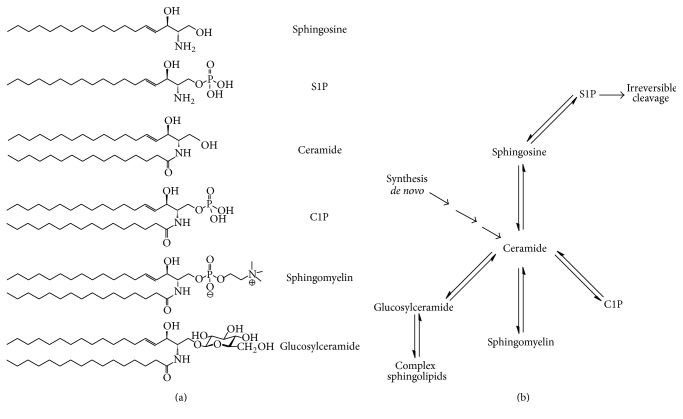
(a) Structure of the main bioactive sphingolipids: sphingosine (Sph), sphingosine-1-phosphate (S1P), ceramide (Cer), ceramide-1-phosphate (C1P), sphingomyelin (SM), and glucosylceramide (GluCer). (b) Schematic representation of the central role of ceramide in sphingolipid metabolism.

Sphingolipids (SLs) are plasma membrane components involved in controlling cellular processes such as proliferation, migration, and apoptosis [5]. Moreover, some bacterial species (*Bacteroides*,* Sphingomonas*, etc.) are capable of synthesizing these lipids [6, 7]. SLs are composed of a sphingoid backbone attached to a fatty acid via an amide bond. The main SLs include ceramide (Cer), ceramide-1-phosphate (C1P), glucosylceramide (GC), sphingomyelin (SM), sphingosine (Sph), and sphingosine-1-phosphate (S1P) (Figure 1(a)). S1P is antagonistic to Cer and Sph, since it promotes cell growth and inhibits apoptosis. The enzymes that interconvert Cer, Sph, and S1P regulate their functions. The metabolism of SLs and the participating enzymes is reviewed elsewhere [8, 9]. However, it is important to mention that Cer has a central role in their metabolism, since it can be synthesized by* de novo* pathway or derived from complex SLs such as SM [9, 10] and glycosphingolipids (Figure 1(b)).

## 3. Cer Plays a Harmful Role in IBD

Cer and related products contribute to varied biological processes as signaling molecules; in addition they are also involved in the development and progression of several human diseases including IBD. The hydrolysis of SM, catalyzed by sphingomyelinases (SMases), is an important source of Cer. However, it can be synthesized by other pathways, which may include the participation of certain microorganisms and cytokines. For instance, the major constituent of the outer membrane of Gram-negative bacteria (lipopolysaccharide or LPS) activates acid SMase in macrophages, which increases Cer content [11, 12]. Once produced, Cer and related lipids participate in inflammatory processes of several tissues, where they stimulate immune cells [13–15] by triggering their mitogen-activated protein kinase (MAPK) pathway [16]. The data shows that IL-1 and Cer augment inflammation via increased eicosanoid production [17, 18]. This possibly contributes to tumor development associated to IBD [19, 20].  Table 1 summarizes both the harmful and beneficial effects of Cer.

It is known that interleukin- (IL-) 1 dose- and time-dependently increases Cer accumulation in intestinal epithelial cells (IEC)* in vitro, *increasing the inflammatory response [21]. The way this IL-1-induced Cer rise modulates the immune response is reported. IL-1 or Cer treatment has no effect on IEC, but a cyclooxygenase- (COX-) 2 inhibitor increases their apoptosis. In addition, IL-1 or Cer increases activation of the nuclear factor *κ*-light-chain-enhancer of activated B cells (NF-*κ*B) in a time and dose dependent manner, by reducing the levels of inhibitor of *κ*-light-chain-enhancer of activated B cells I*κ*B*α* and I*κ*B*β*. The effect requires the degradation of I*κ*B*α* and I*κ*B*β* by the proteasome [19]. IL-1 or Cer augments the production of the antiapoptotic protein B-cell lymphoma- (BCL-) 2, while reducing the expression of several proapoptotic molecules: BCL-2 associated protein X (BAX), BCL-2 homologous antagonist/killer (BAK), and BCL-2 associated death promoter (BAD) [19, 20]. IL-1 or Cer decreases cyclin-dependent kinase inhibitor p21 levels and the number of cells in the G_0_/G_1_ phase of the cell cycle, while augmenting cells in the G_2_/M phase. All these data suggest that IL-1 and Cer enhance survival of IEC by activating COX-2 and NF-*κ*B, which results in reduced proapoptotic protein expression and increased levels of antiapoptotic molecules. Thus, augmented inflammation and reduced apoptosis of IEC may contribute to tumorigenesis in IBD patients.

On the other hand, tumor necrosis factor- (TNF-) *α* and interferon-*γ* induce apoptosis of IEC and also impair their barrier function [21]. These proinflammatory proteins result in Cer production, which may be at least partially responsible for their effects on barrier action. In agreement, exogenous SMase dose-dependently increases IEC permeability* in vitro *[34]. Consistently, SMase treatment diminishes transepithelial resistance and a Cer antibody blocks the augmented permeability caused by platelet activating factor (PAF). Lipid rafts (detergent-insensitive glycosphingolipid-enriched domains) in epithelial cells show high levels of SM, Cer, and cholesterol, plus the tight-junction proteins occludin and claudin-4. In fact, Cer colocalizes with a tight-junction protein. Incubating IEC with exogenous SMase results in a fast elevation of Cer plus a reduction of SM and cholesterol [34]. Thus, the data suggest that proinflammatory stimuli activate SMases, which hydrolyze SM into Cer. Cer accumulates in junctional complexes, reducing their cholesterol levels and provoking their destabilization, which eventually produces a dysfunctional epithelial barrier in the intestine.

In this sense, incubation of a colon cancer cell line with exogenous SMase results in rapid elevation of the mRNA for matrix metalloproteinase- (MMP-) 1 and MMP-10. In fact SMase dose-dependently increases the expression of MMP-1 protein. IL-1*β* and TNF-*α* augment MMP-1 production in colon cancer cells and fibroblasts from healthy subjects and patients with UC. MMP-1 degrades the extracellular matrix and is thought to damage the colonic mucosa. Inhibition of acidic SMase with imipramine blocks the effect of IL-1*β* and TNF-*α* on MMP-1 [22]. Thus, the results suggest that inhibiting acid SMase activity may be a viable therapeutic option for IBD patients (Table 1).

Accordingly, a SMase inhibitor reduces TNF-*α*, IL-1*β*, and IL-6 LPS-induced release from macrophages and diminishes TNF-*α* secretion from human peripheral blood mononuclear cells (PBMC) in response to LPS [23]. The inhibitor also decreases TNF-*α*, IL-1*β*, and IL-6 levels in a colitis animal model produced by administering dextran sulphate sodium (DSS) and reduces the increase in macrophage Cer levels and NF-*κ*B stimulation caused by LPS. This inhibitor also prevents the increase in macrophage acid SMase activity caused by LPS or TNF-*α*. Finally, SM inhibition also increases the viability of cells incubated in media from macrophages exposed to LPS and decreases colon inflammation [23].

The injury caused by Cer is actually caused by its metabolic products, particularly its phosphorylated forms. The harmful Cer derivatives are produced by key enzymes involved in SL metabolism. In this line, the activity of neutral ceramidase (nCDase), an enzyme that catalyzes Cer breakdown, increases in the epithelial layer of the colon after treatment with DSS [30]. The role of nCDase is clearly demonstrated in a null mutant mouse. After DSS treatment, Cer increases in the epithelial layer of the colon in both wild-type and nCDase^−/−^ mice, while S1P concentration decreases in wild-type mice but increases in nCDase^−/−^ animals. Cer only increases in the blood of mutant mice due to DSS, while systemic S1P levels augment in both wild-type and nCDase^−/−^ mice after DSS administration. TNF-*α* and COX-2 expression increase after DSS treatment in the epithelial layer of wild-type and mutant mice, respectively. DSS causes systemic inflammation in both genotypes, as indicated by decreased red blood cells and increased white blood cells; though neutrophils and lymphocytes are higher in nCDase^−/−^ mice. Finally, endotoxin levels are increased in the serum of mutant mice after DSS administration. Thus, nCDase may protect against inflammation, since when it is lacking worse UC symptoms develop [30].

Oral SM ingestion increases SM in feces and IEC of DSS-treated mice [24]. SM feeding may be harmful since it increases weight loss, intestinal mucosal inflammation, and epithelial damage in mice exposed to DSS. Inflammation of the intestinal mucosa is also augmented by dietary SM in IL-10^−/−^ mice. Importantly, SM supplementation results in higher cathepsin D activity and IEC apoptosis [24]. SM feeding increases Cer in control and DSS-treated mice [25]. Similarly, a human colon cell line (HT-29) also converts SM into Cer. Cathepsin D and a proapoptotic protein (BCL-2 homology 3 interacting-domain death agonist or BID) augment in HT-29 cells upon SM treatment. Indeed, SM and DSS activate BID and reduce BCL-2 levels. SM causes apoptosis of HT-29 cells and IEC but phosphatidylcholine protects them. Similarly, SM affects tight-junction proteins making tight junctions weaker, whereas phosphatidylcholine has the opposite effect [25].

## 4. Cer Plays a Beneficial Role in IBD

There are also studies suggesting that Cer and SMases, especially if exogenously applied, may be beneficial for IBD treatment. In this line, it is important to note that their action may depend on how and where Cer is produced. For instance, exogenous acidic and neutral SMases dose-dependently increase Cer levels and trigger NF-*κ*B, mimicking the effect of TNF-*α* [35]. However, the effect of these SMases on NF-*κ*B activation differs in kinetics and the stimulated *κ*B complexes. Acidic SMase turns on p50/p50 homodimers later (20 hours) than neutral SMase, which activates RelA/p52 or RelA/p50 heterodimers at 30 minutes. In fact, IL-8 expression is more than double with neutral SMase compared to acidic SMase. Lastly, the latter SMase induces apoptosis of colon cancer cells* in vitro* but neutral SMase has no effect unless NF-*κ*B is inactive. Therefore, Cer-induced apoptosis may depend on the enzyme that produces it or the site where it is produced [35].

Alkaline SMase is a nucleotide pyrophosphatase/phosphodiesterase family member that breaks down dietary SM [36]. Interestingly, it hydrolyzes PAF* in vitro*, while neutral SMase does not affect PAF [15]. In fact, alkaline SMase activity against SM or PAF is inhibited by high amounts of PAF or SM, respectively. Moreover, its effect on PAF hydrolysis is dose- and time-dependent and is enhanced by bile salts. Low concentrations of zinc (0.1–0.25 mM) stimulate its activity against PAF, while higher levels dose-dependently inhibit PAF hydrolysis. Importantly, PAF incubation with alkaline SMase eliminates its functional effects: p42 and p44 MAPK phosphorylation, IL-8 release, and leukocyte chemotaxis are inhibited [15]. Since PAF displays proinflammatory effects [29, 37], these results suggest that alkaline SMase plays a protective role against the development of IBD and colon cancer. In fact, two reports show that this is indeed the case. Rectal administration of alkaline SMase diminishes DSS-induced inflammation and preserves the colonic epithelium [27]. Similarly, mice lacking alkaline SMase show higher colon tumor incidence and more aggressive cancers due to azoxymethane plus DSS treatment [28].

Oral SM administration may reduce inflammation caused by DSS [31]. Similarly, dietary SLs block tumor development and repress colon cancer [38, 39]. In line, inflammation onset is delayed by SM feeding in mice lacking peroxisome proliferator-activated receptor- (PPAR-) *γ* in epithelial and hematopoietic cells and their recovery is accelerated [40]. Dietary SM increases survival, reduces harmful colonic changes, and diminishes tumor area in PPAR-*γ* ^−/−^ mice. SM supplementation also lowers lymphocyte infiltration into the colon, reduces carcinoma load, decreases F4/80^+^ macrophages in the mesenteric lymph node, and tends to reduce cluster of differentiation (CD)4^+^ T cells in both mutant and wild-type mice. SM feeding augments several chemokines plus their receptors and genes that participate in the differentiation of CD4^+^ T cells towards both proinflammatory and anti-inflammatory phenotypes. Dietary SM is anti-inflammatory by reducing regulatory gene expression and modifying genes involved in tissue protection or regeneration, suggesting that SM feeding may alter tumor development by reducing inflammation [40]. SM may affect inflammatory processes in a PPAR-*γ* dependent manner but its effect on cancer seems independent of this receptor. Thus, SM ingestion may be beneficial or harmful (see above), perhaps depending on its source.

The extracellular action of Cer has shown beneficial effects in the damaged colon, maybe by binding to receptors not related to SLs, such as the leukocyte monoimmunoglobulin-like receptor 3 (LMIR3) [41]. LMR3^−/−^ mice are very vulnerable to colitis induced by DSS, shown by increased weight loss and disease activity as well as reduced colon length and survival [26]. These mutants show greater infiltration of neutrophils, eosinophils, and mononuclear cells into the colon. These cells together with mast cells express LMIR3 on their surface and DSS treatment increases its expression in mast cells. DSS augments the number of mast cells in both genotypes, but this is greater in LMIR3^−/−^ mice. Similarly, degranulated mast cells are also higher in mutant mice. IL-6, IL-17A, and TNF-*α* as well as chemokine transcripts and proteins are increased by treatment with DSS in LMIR3^−/−^ mice. Bone marrow and mast cell transplantation show that the latter cells participate in colitis aggravation in mutant mice. Injury to the colon results in the presence of extracellular ATP which activates P2X7 purinoceptors in mast cells, which release inflammatory molecules [42]. Consistent with this, DSS increases the levels of ATP in the colon. In the absence of Cer, ATP treatment increases mast cell degranulation and secretion of neutrophil chemoattractants such as leukotriene B4, while Cer represses these effects in wild-type but not LMIR3^−/−^ mice. Similarly, Cer inhibits IL-6 synthesis in wild-type mast cells but not in mast cells from mutant mice. Anti-Cer antibodies worsen colitis symptoms in wild-type but not LMIR3^−/−^mice, while Cer liposomes suppress mast cell degranulation in the colon of wild-type mice but not in mutant mice [26]. The data suggest that Cer liposomes may actually be useful as an IBD therapeutic strategy.

## 5. Sphingosine-1-Phosphate and IBD

S1P is synthesized from Sph, a product of Cer breakdown, by two enzymes: sphingosine kinase 1 and sphingosine kinase 2 (SK1 and SK2). Once produced, S1P exerts its action by two mechanisms: directly via intracellular targets or by binding to one of its five different membrane receptors named S1PRs [43]. S1P degradation is regulated reversibly by S1P phosphatases or irreversibly by the S1P lyase enzyme (S1PL). S1P has a significant role in regulating immune cell trafficking, inflammation, angiogenesis, and enhancing cell survival. S1P treatment enhances the survival of B and T cells and inhibits both homoeostatic proliferation and T cell receptor-induced proliferation of T cells, as well as inhibiting cytokine production [44].

It was proposed that S1P favors cell proliferation and survival, as well as inflammation mediated by prostaglandins since it acts as a chemoattractant agent for basophils, neutrophils, and NK cells by upregulating COX-2 and PGE_2_ expression [32, 33, 45, 46]. It is important to mention that the inflammatory response is not limited to the effects of S1P and C1P expression in the intestinal tissue. The resulting PGE_2_ expression can induce the production of interleukins 4, 5, and 10 (Th2 profile) and negatively regulate the expression of interferon *γ*, TNF-*α*, and interleukins 1*β*, 2, and 12 [47]. Th2 profile cytokines can then induce further expression of PGE_2_ and COX-2 in the intestinal tissue, which sustains and increases inflammation. The rise in prostaglandin and COX-2 induces metalloproteinase expression, which, due to the inflammatory conditions that predominate in the intestinal tissue, favor its destruction (Table 1). The principal effect of inflammation on the intestine is the loss of function and structure of the intestinal mucosa and as a consequence failure in the absorption of nutrients, translocation of microbiota bacteria, and changes in the intestinal microenvironment that favor the development of pathogenic bacteria. Thus, S1P has a harmful role in inflammatory illnesses including IBD [48].

Modulating S1P signaling has been proposed as a therapeutic target in IBD treatment [49]. In this line, several studies have been published which use S1PR antagonists in animal models of colitis. Two independent reports show that treatment with FTY20 significantly attenuates the development of colitis induced by DSS or due to genetic deficiency of IL-10. In addition, similar results are obtained with the use of two different antagonists: W-061 and KRP-203 [50–53]. On the other hand, SK1 knockout mice are less susceptible of developing colitis after treatment with DSS [54]. Similarly, administration of SK inhibitors (ABC747080 and ABC294640) reduces the development of colitis induced by DSS in a mice model [55]. Additionally, treatment with a selective S1P1 receptor agonist (SEW2871) improves colitis symptoms in IL-10 deficient mice [56].

## 6. Probiotics Exert Beneficial Actions by Modifying Intestinal Lipids

Our bodies are colonized by trillions of microorganisms from more than 1000 different species. The majority of microbes colonizes the gut, having an important role in nutrition and may be associated with bowel diseases [57]. It has been proposed that dysbiosis (an imbalance in the quantity or type of gut microorganisms) produces or worsens inflammatory diseases. In this sense, diverse therapies using probiotics have been proposed as treatments for IBD [58]. Probiotics are defined as living microorganisms which, when administered in adequate amounts, confer a health benefit on the host [59].

**Figure 2:**
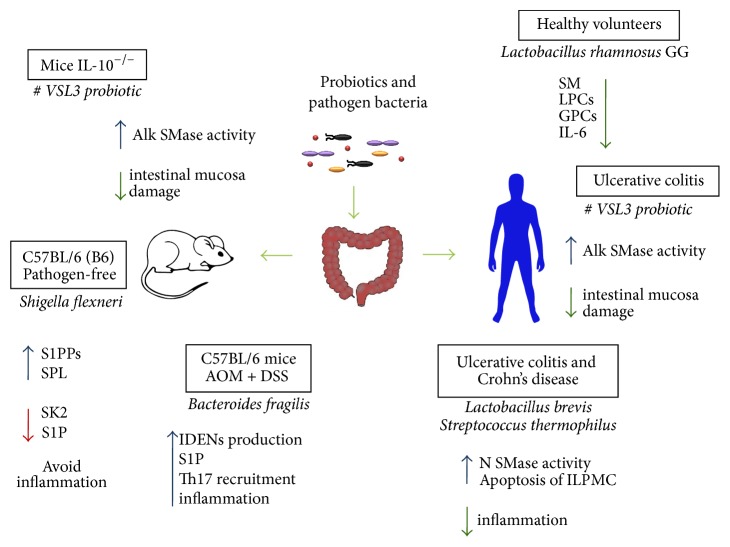
Known effects of pathogenic bacteria, microbiota members, and probiotics on SL signaling in IBD. Probiotics increase SMase activity and diminish intestinal inflammation reducing mucosal damage in both humans and a mouse model.* Bacteroides fragilis,* a known microbiota member, induces inflammation by stimulating epithelial production of IDENs containing high levels of S1P and mediating Th17 recruitment. Conversely, the pathogen* Shigella flexneri* can avoid the inflammatory response by decreasing S1P levels, downregulating SK2 expression and increasing SPL and S1PPs expression. SM: sphingomyelin, LPCs: lysophosphatidylcholines, GPCs: glycerophosphatidylcholines, IL-6: interleukin-6, ILPMC: intestinal* lamina propria* mononuclear cells, IDENs: intestinal derived exosome like nanoparticles, S1P: sphingosine 1 phosphate, SK2: sphingosine 1 phosphate kinase 2, SPL: sphingosine 1 phosphate lyase, and S1PPs: sphingosine 1 phosphate phosphatases.

Probiotics show immunomodulatory actions* in vitro*, in animal models and in humans [60, 61], especially in the context of inflammatory diseases [62]. In this line, treatment with* Lactobacillus rhamnosus* GG causes significant lipid alterations in healthy humans [63]. This treatment increases triacylglycerols but decreases lysophosphatidylcholines (LPCs), glycerophosphatidylcholines, and SMs. These changes correlate moderately with IL-6 levels, especially the diminished LPCs, and may participate in the beneficial effects of this probiotic on intestinal epithelial barrier function [63] as illustrated in Figure 2.

Similarly, a probiotic with eight different bacterial strains has beneficial effects on the intestine [64]. IL-10 knockout mice have lower baseline activity of alkaline SMase compared to wild-type animals and treatment with this probiotic augments its function in the ileum and colon [65]. The probiotic increases alkaline SMase action in wild-type mice and reduces damage to the colonic mucosa in IL-10 knockouts. In humans suffering from UC, the probiotic elevates alkaline SMase function and diminishes disease activity [65]. Thus, the results suggest that augmented activity of alkaline SMase is beneficial in patients with IBD.

Sonicates of probiotic* Lactobacillus brevis* or* Streptococcus thermophilus* show high neutral SMase activity compared to sonicates of nonprobiotic* Escherichia coli* and* Escherichia faecalis *[66].* L. brevis *sonicates cause apoptosis of intestinal* lamina propria* mononuclear cells (ILPMC) from healthy individuals and patients with UC or CD. Interestingly, the effect is more pronounced in the latter. Similarly, sonicates from* S. thermophilus* result in elevated apoptosis of ILPMC from people suffering IBD. Activation of PBMC and ILPMC with antibodies for CD3 and CD28 results in higher apoptosis of these cells in response to the probiotic organisms. Exogenous Cer or neutral SMase also augment apoptosis of ILPMC from healthy subjects and individuals with IBD (Figure 2). Again, the effect is less evident in the former. Both* L. brevis *sonicates and exogenous neutral SMase increase c-jun N-terminal kinase activation in ILPMC.* L. brevis *sonicates cause the production of higher levels of reactive oxygen species in ILPMC. Sonicates of* L. brevis* and* S. thermophilus* incubated with glutathione lose their ability to induce apoptosis of ILPMC from healthy individuals and those affected with CD. In a similar fashion, glutathione abrogates the apoptotic effect of the sonicates on activated PBMC. A specific inhibitor of neutral SMase diminishes apoptosis of ILPMC induced by* L. brevis *sonicates [66]. These results suggest that probiotics generate elevated levels of Cer via their endogenous SMase, which exerts anti-inflammatory effects by killing resident and blood-derived immune cells.

Although the exact mechanism is not completely identified, some bacteria can modulate the host immune system by modulating S1P levels. For example, a study showed that, in a mouse model and a human cell line,* Shigella flexneri* downregulates SK2 expression but upregulates the expression of sphingosine 1 phosphate lyase (SPL) and sphingosine 1 phosphate phosphatases (S1PPs), thus decreasing S1P levels as a mechanism to avoid the inflammatory response [67]. Conversely, secreted particles from enterotoxigenic* Bacteroides fragilis* stimulate intestinal epithelial cells to produce intestinal derived exosome like nanoparticles (IDENs). These IDENs contain elevated levels of S1P, CCL20, and PGE_2_, which mediate Th17 recruitment and induction contributing to intestinal inflammation and cancer [68] (Figure 2).

## 7. Invariant Natural Killer T Cells and Bacterial ***α***-Galactosylceramide

Probiotic bacteria may produce compounds quite similar to *α*-galactosylceramide, which potently activates invariant natural killer T cells (iNKT). These cells are specifically reduced by treatment with azoxymethane followed by DSS, which induces colitis and colon cancer [69]. Mice lacking iNKT cells show more and bigger tumors, as well as worse inflammation indicators than wild-type mice. Azoxymethane/DSS administration augments CD25^+^ CD4^+^ T cells and NK1.1^+^ T cells in mice lacking iNKT cells. The number of IL-13^+^ CD3^+^ cells and IL-13 release in the mesenteric lymph node and colon are increased in these animals. Importantly, *α*-galactosylceramide administration elevates the Th1/Th2 ratio in the mesenteric lymph node, while inflammation and the number of tumors are decreased in the colon. Treatment with *α*-galactosylceramide diminishes the number of colonic NK1.1^+^ T cells and IL-13 release from colonic lymphocytes [69]. The data suggest that probiotics stimulate iNKT cells, which reduce inflammation and development of colon cancer by altering T cell populations and cytokine secretion.

**Figure 3:**
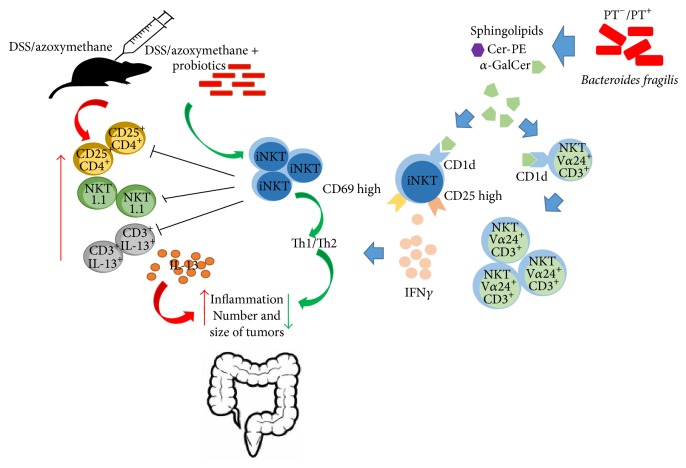
Bacterial *α*-galactosylceramide as iNTK regulator in colon cancer. Treatment with azoxymethane followed by DSS reduces the number of iNTK cells and augments CD25^+^ CD4^+^ T cells, NKT1.1^+^ T cells, and IL-13^+^ CD3^+^ cells, as well as IL-13 release. All this contributes to form a greater number of tumors of a larger size. Probiotic bacteria can produce compounds similar to *α*-galactosylceramide, which prevents inflammation and reduces the number and size of tumors.* B. fragilis* produces *α*-galactosylceramide which stimulates iNKT cells binding to CD1d, increasing production of IFN-*γ* and proliferation of CD3^+^ V*α*24^+^ cells. DSS: dextran sulphate sodium, iNKT: invariant natural killer, NKT: natural killer T cell, and PT: serine palmitoyltransferase.

An investigation of the SLPs synthesized by a prominent constituent of the gut microbiota was performed [70]. A putative serine palmitoyltransferase, which catalyzes the first committed step of sphingolipid biosynthesis, was deleted from* Bacteroides fragilis* NCTC 9343. No double mutants are obtained, suggesting that the enzyme is critical for survival. Single mutants lack sphingolipid production, allowing identification of the sphingolipids produced by wild-type* B. fragilis*. These bacteria synthesize Cer phosphorylethanolamine, its matching dihydroceramide analog, and *α*-galactosylceramide. The latter binds to CD1d to activate mouse and human iNKT cells, as shown by IL-2 and IFN-*γ* synthesis. This lipid also stimulates human PBMC, as shown by proliferation of CD3^+^ V*α*24^+^ cells, and activated iNKT cells* in vivo*, evidenced by higher CD25, CD69, and IFN-*γ* expression (Figure 3). Moreover the effect is blocked by CD1d antibodies [70]. The results suggest that an important member of the gut microbiota produces *α*-galactosylceramide, which stimulates PBMC and iNKT cells.

Another study suggested that *α*-galactosylceramide did not activate iNKT cells, but instead it reduced their stimulation both* in vitro* and* in vivo* by competing for CD1d binding or impeding detection by iNKT cells [71]. Mice monocolonized with* B. fragilis* that lack their presumed serine palmitoyltransferase have a higher number of colonic iNKT cells and worse colitis symptoms, evidenced by a more pronounced weight loss as well as higher IL-4 and IL-13 secretion. The iNKT cell population is reduced by neonatal treatment with a CD1d antibody; this blocks oxazolone-induced colitis in adult mice with altered* B. fragilis*. These animals have similar colonic bacteria number, comparable chemokine levels, analogous iNKT cell stimulation, and apoptosis plus similar polysaccharide A expression. The bacterial SLs diminish iNKT proliferation, but only when mice were exposed to them prenatally. Ceramides, glycosylceramides, and phosphoethanolamine ceramides are present in wild-type but not in mutant* B. fragilis*. iNKT cell activation is decreased by glycosylceramides, including *α*-galactosylceramide. Importantly, *α*-galactosylceramide treatment during the neonatal period diminishes the number of colonic iNKT cells and improves oxazolone-induced colitis in mice with altered bacteria [71]. Thus, *α*-galactosylceramide produced by a prominent constituent of the gut microbiota is beneficial. The issue of whether this lipid stimulates or inhibits iNKT cells remains to be resolved.

Glycosylceramides contained in mammalian milk and serum activate iNKT cells; indeed human iNKT cells are stimulated by cow and human milk [72]. But glycosylceramides from the spleen of Gaucher's disease patient do not affect iNKT cells. This suggests that an unknown ingredient in mammalian milk and serum is responsible for the observed effect because the glycoceramides within cow's milk and Gaucher's spleen are comparable. Upon further analysis, the ingredient is likely *α*-galactosylceramide which is also present in the thymus of mammals and activated iNKT cells [72]. The most probable explanation is that mammalian microbiota is responsible for producing this *α*-galactosylceramide, although the intriguing possibility that it can be synthesized by mammals cannot be dismissed. Of note, in this study galactosylceramides stimulate iNKT cells as explained in Figure 3.

## 8. Conclusion

IBD has become an emergent public health problem. As discussed previously, although its exact etiology remains unclear, the role of SLs in contributing to the inflammatory process is evident. Indeed, besides its role in digestive function, the gut is actually considered as an immune organ. This is in large part due to the signaling molecules that are produced within it, particularly those comprised by SLs, which affect not only the intestinal tract but the whole immune system. As mentioned above, Cer is a central molecule in defining the role of the immune response through its different metabolic byproducts: SM, Sph, and S1P, which mediate specific responses. SMases are of particular interest because of their differential activities. Thus, research on therapeutic agents able to modulate SMases and formulations for tissue-specific delivery is mandatory. The conversion of Cer into different SLs, which enhance or prevent the inflammatory response, depends on cell molecular signals and the cell microenvironment. In this sense, gut microbiota may provide the conditions that define the source and fate of SLs as modulators of the immune response. For instance, microorganisms provide enzymes or their activators to promote Cer metabolism and thereby regulate cytokine production. Thus, gut microbiota is a new and attractive target for the control of IBD and even other inflammatory conditions.

In this sense, prebiotic and probiotic agents emerge as pivotal players in the control of the immune response in this organ. Further therapies may include the preservation of natural microbiota in the bowel in order to avoid IBD and new therapies based on nutritional programs including food that favors beneficial species or even seeding the intestinal tract with strains that can produce specific SLs, depending on the pathological situation. These promising possibilities deserve further scientific and clinical investigation.

## Figures and Tables

**Table 1:** Potential harmful and beneficial mechanisms of Cer and S1P in IBD.

SLs	Effect	Target	Tissue/cells	References
Harmful				

Cer	Increases	NF-*κ*B & COX-2	Intestinal tract	[12, 13]
		BCL-2 expression	Intestinal tract	[12, 13]
		Inflammation	Intestinal tract	[12, 13]
	Decreases	Apoptosis	Intestinal tract	[12, 13]
		Expression of BAX, BAK & BAD	Intestinal tract	[12, 13]

Cer	Activates	Immune cells	Intestinal tract	[14–16]
		MAPK cascade	Intestinal tract	[17]
	Decreases	Cholesterol levels	IEC tight junctions	[20, 21]
		Barrier function	IECs	[20, 21]

SMase	Increases	TNF-*α*, IL-1*β* & IL-6 secretion	Intestinal macrophages	[22]
		LPS-induced TNF-*α* release	PBMCs	[22]
		TNF-*α*, IL-1*β* & IL-6 levels in DSS-induced colitis	Colon	[22]
		LPS-induced increase of Cer & NF-*κ*B	Intestinal macrophages	[22]
		Inflammation	Colon	[22]
		LPS-induced cell death	Intestinal tract	[22]

nCDase	Decreases	DSS-induced S1P and COX-2 levels	Colon	[23]
		Endotoxin levels	Serum	[23]

Dietary SM	Increases	DSS-induced inflammation	Colon	[24, 25]
		Cathepsin D activity, BID activation, HT-29 cell & IEC apoptosis	Colon	[24, 25]
	Decreases	BCL-2 levels	Colon	[24, 25]

Beneficial				

Cer	Decreases	IL-6 synthesis Mast cell degranulation	Colon	[26]

Alkaline SMase	Decreases	PAF	Intestinal tract	[16]
		DSS-induced inflammation	Rectum	[27]
		Tumor incidence	Colon	[28]
		DSS + azoxymethane cancer aggressiveness	Colon	[28]
	Protects		Colonic epithelium	[29]

Acidic SMase	Increases	Apoptosis	Colon cancer cells	[30]

Dietary SM	Decreases	DSS-induced inflammation	Colon	[29]
		Lymphocyte entry	Colon, PPAR-*γ* ^−/−^ mice	[31]
		Carcinoma burden	Colon, PPAR-*γ* ^−/−^ mice	[31]
		F4/80^+^ macrophages	Mesenteric lymph node	[31]
	Delays Hastens	Inflammation	Intestinal tract	[31]
		Recovery	Intestinal tract	[31]
	Increases	Survival of PPAR-*γ* ^−/−^ mice		[31]
		Chemokines and their receptors	Intestinal tract, PPAR-*γ* ^−/−^ mice	[31]
		CD4^+^ T cell maturation genes	Intestinal tract, PPAR-*γ* ^−/−^ mice	[31]

S1P	Increases	B & T cell survival	Intestinal tract	[26]
	Decreases	T cell proliferation Cytokine synthesis	Intestinal tract	[26]

S1P	Increases	COX-2 & PGE_2_, inflammation, metalloproteinase production	Intestinal tract	[32, 33]
