# Corrigendum to “Sphingolipids as Mediators in the Crosstalk between Microbiota and Intestinal Cells: Implications for Inflammatory Bowel Disease”

**DOI:** 10.1155/2016/7267956

**Published:** 2016-11-14

**Authors:** Bryan Phillips-Farfán, Karla Carvajal, Edgar Alejandro Medina-Torres, Sara Elva Espinosa-Padilla, Gemma Fabrias, Luz Camacho

**Affiliations:** ^1^Laboratorio de Nutrición Experimental, Instituto Nacional de Pediatría, 04530 Ciudad de México, Mexico; ^2^Unidad de Investigación en Inmunodeficiencias, Instituto Nacional de Pediatría, 04530 Ciudad de México, Mexico; ^3^Research Unit on Bioactive Molecules, Department of Biomedicinal Chemistry, Institute for Advanced Chemistry of Catalonia (IQAC-CSIC), 08034 Barcelona, Spain

In the article titled “Sphingolipids as Mediators in the Crosstalk between Microbiota and Intestinal Cells: Implications for Inflammatory Bowel Disease” [1], reference [28] was incorrectly added as follows:

[28] “Y. Chen, S.-C. Xu, and R.-D. Duan, ‘Mevalonate inhibits acid sphingomyelinase activity, increases sphingomyelin levels and inhibits cell proliferation of HepG2 and Caco-2 cells,' Lipids in Health and Disease, vol. 14, no. 1, article 130, 2015.”

It should be replaced with the following reference:

[28] “Chen Y, et al. Enhanced colonic tumorigenesis in alkaline sphingomyelinase (NPP7) knockout mice. Mol Cancer Ther, 14:1, 259-67, 2015.”
